# Toward Sci-**φ**: A Lightweight Cloud PaaS for Developing Embarrassingly Parallel Applications Based on Jini

**DOI:** 10.1155/2014/526953

**Published:** 2014-02-13

**Authors:** Patrizio Dazzi

**Affiliations:** ISTI-CNR, 56124 Pisa, Italy

## Abstract

Embarrassingly parallel problems are characterised by a very small amount of information to be exchanged among the parts they are split in, during their parallel execution. As a consequence they do not require sophisticated, low-latency, high-bandwidth interconnection networks but can be efficiently computed in parallel by exploiting commodity hardware. Basically, this means cheap clusters, networks of workstations and desktops, and Computational Clouds. This computational model can be exploited to compute a quite large range of problems. This paper describes Sci-*φ*, an almost complete redesign of a previous tool of ours aimed at developing task parallel applications based on Java and Jini that were shown to be an effective and efficient solution in environments like clusters and networks of workstations and desktops.

## 1. Introduction

Parallel computing, in a nutshell, is a form of computation in which many elaborations are performed simultaneously. It is based on the principle that many large problems can be solved by dividing them into smaller ones, which are then solved concurrently to spend less time than their sequential execution requires. The resulting gain is called *speedup*. Basically, it consists in an indication on how much a parallel algorithm is faster than the corresponding, equivalent, sequential algorithm. Speedup (*S*) is computed as

(1) $$\begin{array}{c} \;S_{P}=\frac{T_{1}}{T_{P}}, \end{array}$$

where *T*_1_ is the execution time of the sequential algorithm, *P* represents the number of processors used for the computation, and *T*_*P*_ is the time requested by the parallel algorithm executing on *P* processors.

The most favourable scenario is the one in which the speedup deriving by the parallel execution of a program is linear (the exploitation of *n* processing elements reduces the computation time by a factor of 1/*n*). Unfortunately, only a few problems achieve the optimal speedup. In fact, many parallel solutions and applications show at most a near-linear speedup and only for small numbers of processing elements. From a formal point of view, the (maximum) speedup that an algorithm can provide on a parallel computing platform is given by *Amdahl's law*, which has been originally formulated by Amdahl in the 1960s [1]. Such law states that the overall speedup achievable through parallel execution is limited by the portion of the computation that can not be run in parallel. Given a program, let us consider *α*, the time portion to spend for the computation of parts that can not be run in parallel; then

(2) $$\begin{array}{c} \underset{P\to \infty }{\lim}\frac{1}{\left(\left(1-\alpha \right)/P\right)+\alpha }=\frac{1}{\alpha } \end{array}$$

is the maximum speedup that can be achieved by computing the whole program in parallel.

This defines an upper limit on the convenience of adding more and more parallel execution units for reducing the completion time of a program.

Besides the parts that can not be executed in parallel, another key aspect to deal with when implementing parallel applications consists in the dependencies between program's data. In short, no program can run faster than its critical path (basically, the longest chain of dependent calculations), since computations depending on previous calculations must be executed in order. As an example, consider *P*_*i*_ and *P*_*j*_ to be two program segments. Bernstein's conditions [2] specify in which cases they are independent and, as a consequence, can be executed in parallel. For *P*_*i*_, let *I*_*i*_ be all of the input variables, and *O*_*i*_ the output variables, and likewise for *P*_*j*_.

We can state that *P*_*i*_ and *P*_*j*_ are independent segments if they satisfy the following three conditions:

(3) $$\begin{array}{c} I_{j}\cap O_{i}=\emptyset ,\\ I_{i}\cap O_{j}=\emptyset ,\\ O_{j}\cap O_{i}=\emptyset . \end{array}$$

If one of the first two conditions is violated, then flow dependencies exist. Thus, one of the segments is expected to produce data that will be needed by the other segment. As a consequence, they cannot be run in parallel. The third, and final, condition represents an output dependency. It means that when two segments write to the same location, the result finally stored comes from the logically last executed segment [3].

In a sense, by using a geometric metaphor we can state that Amdahl's law and Bernstein's conditions define kinds of horizontal and vertical bounds to parallelism exploitation. The former indicates the maximum “width” that a (segment of *a*) parallel application can assume; the latter defines the minimum “height” of the critical path, namely, the steps to be conducted in a sequential manner to compute an application. In fact, according to Amdahl's law, parallel applications are characterised by a very low value of *α* scale almost linearly, allowing a very wide width. In addition, when Bernstein's conditions indicate a significant independency between the code segments composing the program, the height of the parallel application is low and the computation of most of its parts can be performed concurrently.

According to our introduced metaphor, if a problem can be solved by a program whose shape is wide and low, it means that it can be computed in parallel without a particular effort, by separating it into a number of tasks to compute in parallel. This kind of problems is generally referred to as an embarrassingly parallel problems. Often they can be implemented by programs whose tasks have no dependencies on each other. Such tasks tend to require little or no communication of results and are thus different from more complex computing problems that may require frequent information exchange, for example, the communication of intermediate results.

Parallel applications realised according to this model are easier to implement than more complex kinds of parallel applications; they also do not require high-bandwidth, low-latency, and expensive communication infrastructures to scale-up when the number of resources involved in the computation increases significantly. Typically, this kind of applications can be run on commodity based clusters, networks of workstations, and, more recently, Clouds and Federation of Clouds. Basically these environments consist of infrastructures that allow dealing with a huge amount of computational resources but are usually characterised by a limited range of guarantees on network subsystems [4–6] as well as on the limited reliability of the hardware exploited. Anyway, in spite of the quite simple structure of this parallel paradigm, several different kinds of applications can be effectively and efficiently implemented by structuring them according to it.

Examples include distributed set processing of queries in distributed relational databases, web-servers, several types of fractal calculations (basically all the ones where each point can be calculated independently), brute-force searches in cryptography, large scale image recognition software, computer simulations comparing many independent scenarios (e.g., climate models), genetic algorithms as well as other evolutionary computation metaheuristics, numerical weather prediction, and simulations of particle physics.

As can be noticed by reading the above list, there exist some classes of scientific-related applications that can be approached by exploiting this paradigm. Indeed, not having any particular requirement in terms of data exchange, embarrassingly parallel problems can be computed on server farms built with commodity hardware, which do not require any special communication and data storage infrastructure as supercomputers require instead. In a previous paper of ours we presented JJPF [7], a parallel programming framework, whose main features are recalled in Section 2. Basically, it is a tool for implementing stream parallel applications, written in Java and exploiting Jini [8] for resource discovery and job assignment.

JJPF provides some useful features, like

- *automated load balancing* across the computing elements participating in the computation;
- *automatic resource discovering and recruiting* exploiting standard Jini mechanisms;
- *automated fault tolerance* achieved by substituting faulty resources with other ones (if any) in a seamless and automatic way.

Anyway, JJPF has been designed before the Cloud-era and also before multicore microprocessors became part of standard configurations of commodity hardware. As a consequence, it is unable to properly exploit the performance gain derivable from actual CPUs. In addition, it cannot be configured in a modular fashion to let users and system administrators manage and enforce the partitioning of the available resources among applications that are actually running in the same machine. This is a fundamental requirement to realise a multitenancy Platform as a Service (PaaS) exploiting a private Cloud for user computations.

In this paper we present Sci-*φ*. It has been designed to overcome the JJPF limitations and to be exploited by multiple tenants at the same time in private Clouds. Sci-*φ* also allows partitioning the application workload in a more efficient way and exploiting multicore microprocessors.

The remainder of this paper is structured as follows. Section 2 describes our former contribution and the underneath technology for resource discovery: Java Jini. Then, Section 3 introduces Sci-*φ*, our proposed next generation framework that enhances JJPF to make it suitable to be used as a simple and lightweight Cloud PaaS. Later, in Section 4 are presented the experiments we conducted to give a preliminary evaluation of Sci-*φ*. Similar approaches existing in literature are presented in Section 5. Finally, in Section 6 we draw our final considerations about this work and we present our thought on the future work that we plan to conduct in the field.

## 2. JJPF: Our Former Solution

Our former solution consists of a tool for developing task parallel applications, written in Java and based on Jini. JJPF is a framework essentially structured according to a master-slaves organisation. The JJPF master is able to find and to recruit in networks and clusters of workstations a set of available resources to enlist as computing slaves. In order to be exploited by the JJPF master, these machines have to run a proper system daemon behaving as a computation service; it does support the execution of stream applications. In short, it can be stated that JJPF enacts and exploits a network-based remote execution service. This is made possible by the underneath network layer based on Jini. Basically, it consists in a network-based approach for the organisation of distributed systems in the form of modular cooperating services.

### 2.1. Jini

Sun Microsystems introduced Jini in July 1998. Essentially, it is a network-centric computational architecture that has been conceived to be a support for “spontaneous networking.” The Jini approach follows the concept behind the motto that lasted long under the Sun Microsystems mark “network is the computer.” In fact, by using Jini, users can plug printers, storage devices, speakers, and, potentially, any kind of device directly into a network. Other computer devices and users connected to the same network are, consequently and automatically, informed that such devices have been added and, hence, are available. Each network device, to be found, has to define itself by means of a specific interface and properly inform an ad hoc designed network device registry.

When someone (or something) decides to use, or to access, a resource belonging to the same network, the Jini support will lead her (or it) to automatically download proper software, which in turn will make possible the interaction with the resource and communicate with it without any particular effort or awareness requirements to the operating system, which indeed does not require having any particular software driver preinstalled. Basically, the “Jini promise” has been enabling hardware manufacturers to make devices that can be attached to a network and used independently operating systems and the other devices connected to such network. This is possible by virtue of the Jini communication and interaction model, which is based on customisable software network proxies.

From an operative point of view, a resource, to be found and used, has to register its proxy in a specific network directory service called “*LookupService*.” Such service is then contacted by clients, who want to exploit the resources available in the network, to retrieve the specific software proxies required for enacting the network interconnection with the devices. The Jini software architecture consists of four software stacked layers:

- directory service;
- JavaSpace;
- Remote Method Invocation (RMI);
- boot, join, and discover protocol.

**Figure 1:**
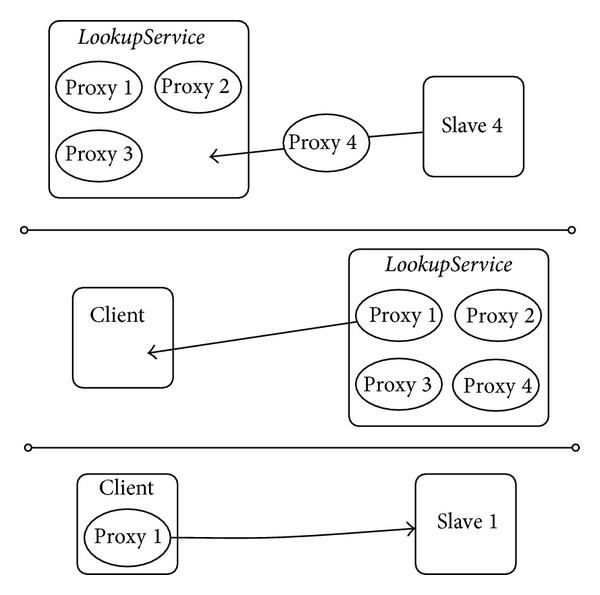
Jini registration and discovery.

When a device is plugged into a Jini-enabled network it register its own proxy into the *lookupService* using the API of the directory service layer. Once it complete this step, it becomes a member of the network. The Java classes needed to let other devices to use it are put in the JavaSpace layer to ease their localisation and download; operations that take place via RMI. Finally, the bottom layer of Jini is devoted to provide low-level boot, join and discovery mechanisms.  Figure 1 depicts the support provided by Jini to device registration, discovery and exploitation.

### 2.2. The JJPF Approach

As we already mentioned, JJPF benefits from the features provided by Jini to build up a remote computing service that can be exploited for the computation of task parallel applications. Jini is exploited by JJPF in a twofold manner: by clients for finding computational resources to run the tasks composing their parallel computations and by slaves (servers) to publish themselves in such a way that they can be found by clients, and, therefore, to receive tasks to elaborate.

Algorithm 1 shows the steps conducted by a JJPF computing slave to be found and used by clients. More in detail, when a slave does start, it searches the Jini discovery service, the *LookupService*. Then, it enters in a loop that terminates only when the slave is turned off or reassigned to other duties. The first step conducted inside the loop consists in the registration into the *LookupService*. This step allows clients to find the actual slave. After the registration, the slave waits for requests coming from clients. When a request arrives, the slave unregisters itself from the directory service to avoid other clients to find it. Then, it starts to compute the tasks received from the client it has been assigned to. If on the one hand this choice simplifies the resources management, it can potentially limit the exploitation of JJPF in multitenants scenarios. After the completion of the computation, it can either terminate its activity or restart the main loop.

On the other side, the activity conducted by clients for finding and exploiting computing slaves is shown by Algorithm 2. This algorithm, like the previous one, starts with the discovery of the *LookupService*. Once it has been found, the client queries for the available slaves. If they are available the client recruits each of them by instantiating an ad-hoc *ControlThread* whose aim is to “drive” the interaction with the slaves just found. This means to assign tasks, by fetching them from the client-defined task vector, to retrieve the results once computed, and to store them in the results vector. In case of faults, *ControlThread* has to signal the problem to the JJPF client runtime that will reassign the tasks previously sent to the faulty slave. Then the main client program waits until all the tasks have been processed and eventually terminates the computation. If no slave is available, the client registers a special object, an *observer*, into *LookupService*. By means of this object the client is alerted if any slave registers itself into the directory service, so that the client can enlist such computational resource. Basically, this means that JJPF uses two distinct mechanisms to support clients in recruiting slaves. One is synchronous and the other one is asynchronous (in fact consisting in a sort of publish-subscribe approach). The synchronous mechanism directly queries *LookupService* about the available slaves, that is, the computational resources that JJPF uses for computing tasks. The asynchronous mechanism works by registering into *LookupService* an *observer* object that will alert the client when new services become available, so that they can be recruited.

JJPF also achieves automatic load balancing among the recruited services. This is possible by virtue of the scheduling approach adopted in the *ControlThreads* managing remote services. Each *ControlThread* fetches tasks to be delivered to the remote nodes from a centralised, synchronised task repository.

As we mentioned above JJPF also automatically handles faults in service nodes. More in detail, it takes care of the tasks assigned to a slave so that if the node does not respond any more they can be rescheduled to other service nodes. This is possible because the only kinds of parallel applications that are supported in JJPF are the ones relying on stream parallel computations. In this case, there are natural *descheduling points* that can be chosen to restart the computation of one of the input tasks, in case of failure of a service node. A trivial choice for the point is the start of the computation of the task. Provided that a copy of the task is kept on the client side, the task can be rescheduled as soon as the control thread notices that the corresponding slave has been disconnected or it is not responding. This is the choice we actually implemented in JJPF, inheriting the design from muskel [9, 10].

One of the key advantages deriving from the usage of JJPF is that all the activities described in the above presented algorithms are automatically performed by the JJPF run time support. No code dealing with service discovery or recruiting is to be provided by application programmers.

Finally, also the installation is easy and nondisruptive regarding the installed software and the system configuration. Indeed, in order to use JJPF on a workstation network or cluster, just the following three steps have to be performed:

1. Jini has to be installed and configured,
2. JJPF services have to be started on the machines that will be eventually used to run the JJPF distributed server,
3. a JJPF client such as the one sketched above has to be prepared, compiled, and run on the user workstation.

### 2.3. The Programming Model of JJPF

To write an application able to exploit the remote execution service realised by JJPF, programmers need to implement a JJPF client and structure it as a kind of stream processor in which the stream items consist of the set of tasks to compute. The tasks composing the applications are executed by the distributed slaves recruited.

The JJPF clients are essentially structured according to the task farm pattern. Programmers only need to write two lines of code to define: which parallel computation to perform, the input data and the output data.

Consider

 
BasicClient cm =
 
new BasicClient (program,
 
null,
 
input,
 
output);
 
cm.compute();

input and output are collections of input and output tasks, respectively. program is an array hosting the code that slaves have to compute on their sides. The code consists of a Class object describing the user worker code. Such code must implement a ProcessIf interface. This interface requires that three methods were implemented: one to provide the input task data (void setData (Object task)), another one to retrieve the result data (Object getData()), and, finally, a method to compute results out of task data (void run()).

## 3. Sci-*φ*

In spite of the several advantages coming from the exploitation of JJPF, for running stream parallel applications on clusters and networks of workstations, it is not really appropriate for current computational environment based on commodity hardware. In addition, with the advent of Cloud Computing, people want more and more to interact with computational resources in a way that recalls the one provided by widely adopted PaaS, just to mention a few, a support for multitenants, a security support (including both authentication and authorisation), a standard way for expressing resources features and task requirements, and so forth.

To this end we propose Sci-*φ* a complete redesign of JJPF to let it address the current typical requirements of such kind of infrastructure via a PaaS approach but preserving the original idea of JJPF. Basically, our aim is to provide a full-featured PaaS for high throughput computations, mainly targeting embarrassingly parallel applications, able to deal with churning machines and that can be nonintrusively installed on clusters and networks of workstations made of commodity hardware.

In the following of this section we summarise the requirements that a next generation tool like Sci-*φ* should provide along with the actual limitations of JJPF.

### 3.1. Modern Commodity Hardware

JJPF has not been designed for exploiting machines equipped with modern CPUs, like multicores. In fact, each machine is registered once into the directory service independently of the number of processor cores or processors onboard. In addition, the JJPF client assigns to each slave machine just a task at-a-time, independently of the number of cores installed into the slave machine.

### 3.2. Classification of Computational Resources

In JJPF the resources register themselves into the directory service without specifying any of their own features. As a consequence, user cannot specify any requirement about the desired computational resources. What a user obtains is just a kind of best-effort computational support.

### 3.3. Security

JJPF does not provide any kind of security support, neither for authentication aspects, nor for authorisation related stuff. This was pretty acceptable when it was firstly adopted but should be introduced now. In particular if it would be used as a Cloud PaaS exploiting resources from multiple Clouds. Indeed, this is a definitely useful aspect in Cloud oriented scenarios, as also suggested by Takabi et al. [11].

### 3.4. Task Description

In JJPF each task needs to implement an ad hoc interface; this is a pretty simple but limited solution. By following this approach existing programs to run with JJPF need to be specifically refactored. If on the one hand this was almost acceptable when JJPF has been designed (i.e., early 2004, when even the Java Callable interface was not yet available) now something more widely adopted should be exploited, for instance, Java EE components to integrate exploiting dependency injection [12] approaches. Another interesting possibility would be the adoption of the Batch Applications support of Java EE 7 [13] that defines a batch programming model, a job specification language, and a batch runtime.

### 3.5. Service Orientation

JJPF is a computing framework in the strict sense of the term. Applications exploiting it need to be actually integrated with it. A better approach would be to make it just a service running on one of the machines it recruits for a computation. This would introduce the typical advantages in migrating from application to services [14], for example, intrinsic interoperability, flexibility, and so forth.

### 3.6. REST Interfaces

To run a JJPF client on a machine, the user needs to have a shell access to such machine. This makes the approach not flexible enough to be exploited by a dynamic range of users. Indeed, each new user requires the intervention of the system administrator for creating an account on such machine. This model should be overcome, for instance, providing widely adopted standard for program execution in Cloud systems, nowadays mainly based on REST interfaces [15].

### 3.7. Task Dependencies

JJPF can be used only for task parallel applications no task dependency is allowed. This limits the kinds of applications that can be computed with it. A more careful design of the client would allow also the computation of applications in which tasks have dependencies on each other. For instance such dependencies can be defined by means of workflow-based solutions or Macro Data Flow approaches [16].

### 3.8. Enhanced Communication Subsystem

JJPF exploits the RMI subsystem embedded in Jini (JERI [17]) both for localising and recruiting slaves as well as for communicating data packages and tasks to compute. If on the one hand this is perfectly in line with the Jini philosophy, on the other hand it is not a particularly efficient solution when the data to transfer increases. An alternative would be to couple RMI with other protocols that are more suitable for high performance data transfer.

#### 3.8.1. The Actual Implementation of Sci-*φ*

As we stated above, Sci-*φ* has been conceived and designed to overcome JJPF and to address all the aforementioned requirements. Its design is modular and flexible, well suited for incremental implementation of the features required. Anyway, even if the design of Sci-*φ* is essentially complete, so far only a reduced set of the requirements mentioned above have been fully addressed. More in detail, we successfully included the support for multicore microprocessors, as we show in Section 4 presenting the experimental evaluation. Moreover, also a way to classify resources has been introduced. In order to make this solution effective we give a resource representation that fundamentally has been inspired by the one used by Amazon for EC2 instances.

We are currently working on the development of other features. Anyway, the current set of implemented features allows conducting a preliminary evaluation, that we described in the next section, and clearly shows that Sci-*φ* provides a notable gain with respect to JJPF in terms of performance on current generation hardware.

## 4. Preliminary Evaluation

Exploiting the actual, prototypal implementation of Sci-*φ* we conducted a preliminary evaluation by developing a program devoted to the computation of the Mandelbrot set and running it both using Sci-*φ* and JJPF. The experiments have been conducted by using two clusters owned by our institution.

### 4.1. The Test Environment

We tested our proposed framework using two different clusters: Novello and Cannonau. The first one, Novello, consists of six machines, each one equipped by a Xeon Quad-core processor (E5520), running at 2.27 Ghz and sixteen Gigabytes of RAM. The second one consists of eight machines, each one equipped with two (quite old) Xeon processors running at 2.0 Ghz and one Gigabyte of RAM. Both clusters interconnect their machines using a Gigabit switch. All the machines composing the clusters run Linux Ubuntu Server Edition.

### 4.2. The Test Application

The application used for our tests is the well-known Mandelbrot Set. Basically, it consists in a figure composed by a set of points whose boundary is a two-dimensional fractal shape. The set is closely related to Julia sets and is named after Benoit Mandelbrot that made it very popular. Mandelbrot set images are made by complex numbers. For each point a particular computation determines if the result tends towards infinity after a certain number of iterations. Treating the real and imaginary parts of each number as image coordinates, pixels are coloured according to how rapidly the sequence diverges, in case it does.

### 4.3. The Results Obtained

**Figure 2:**
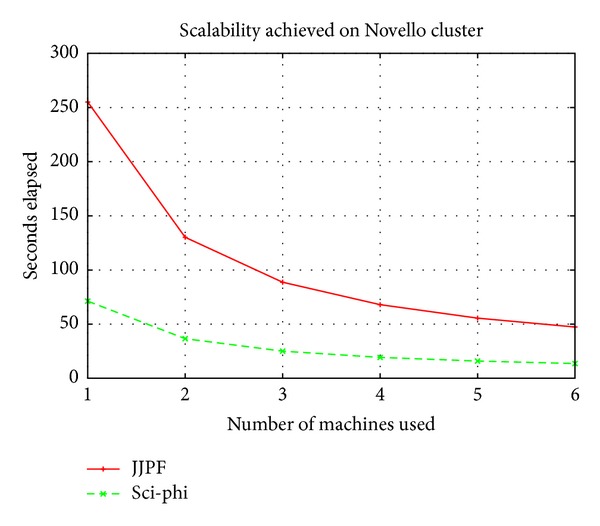
Scalability measured on Novello cluster.

We conducted our evaluation with our two clusters by obtaining encouraging results. In both clusters Sci-*φ* obtained a very good scalability.  Figure 2 shows the results we measured by computing the Mandelbrot Set on Novello cluster. The sequential execution of our test application on a single machine in this cluster required about 251 seconds. As it can be observed, JJPF, that exploits just a core for each machine, provides pretty good scalability results when the number of enlisted machines increases. Our new framework Sci-*φ* is also able to successfully exploit all the cores installed in each machine and maintains the ability of JJPF to scale when additional machines are recruited for the computation.

**Figure 3:**
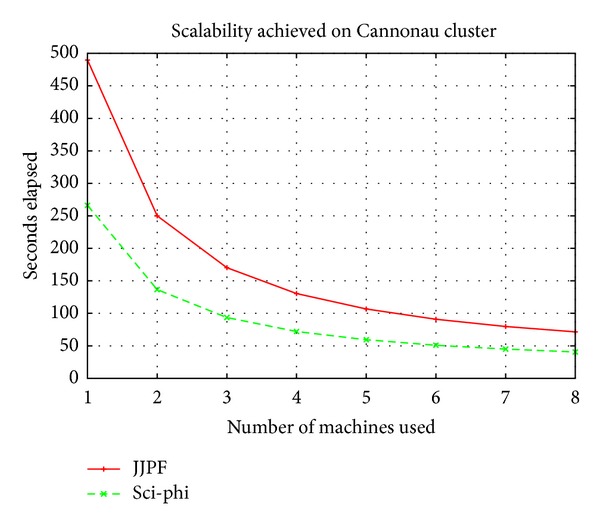
Scalability measured on Cannonau cluster.

Figure 3 shows the results we achieved with the cluster Cannonau. It is an older set of machines but already characterised by an internal parallel degree. Indeed, each Cannonau machine has two processors installed making it a good candidate for our evaluation. In this case the sequential time for computing the Mandelbrot Set was around 478 seconds. This consistent difference with the sequential time obtained with Novello machines can not be imputed to the slight difference in the processor frequency. The interpretation we gave to these phenomena is instead related to the newer processing architecture of Novello's CPU as well as the faster memory it is equipped with.

Regarding the parallel processing time, also in this case it can be noticed that while JJPF is able to scale when the number of machines raises, Sci-*φ* successfully exploits the available resources in each machine to lower the time required to compute the Mandelbrot Set.

## 5. Related Work

Besides JJPF, our Sci-*φ* environment is not our first proposal for providing a stream parallel computing environment. Another of our previously developed parallel programming environments, muskel, already provides automatic discovery of computational resource in the context of a distributed workstation network. Anyhow, in muskel the discovery was simply implemented using multicast datagrams and proper discovery threads. The muskel environment also introduced the concept of *application manager* that allocates computational resource to tasks and provides an autonomic application control in such a way that optimal resource allocation can be dynamically maintained upon specification by the user of a *performance contract* to be satisfied [9, 10].

Several other researchers proposed or currently propose environments supporting stream parallel computations on workstation networks and clusters. Among the others, we mention Cole's *eskel* library running on top of MPI [18], Kuchen's C++/MPI skeleton library [19], and CO_2_P_2_S from the University of Alberta [20]. The former two environments are libraries designed according to the algorithmic skeleton concept. The latter is based on parallel design patterns. Other notable solutions for running embarrassingly parallel tasks are the BOINC [21] project that is a widely used approach for volunteer computing as well as the Condor project [22] that pioneered using the idle time of organisational workstations to do parallel computing.

## 6. Conclusions and Future Work

In this paper we presented Sci-*φ*, an integrated solution supporting the execution of embarrassingly parallel application on cluster or networks of workstations. Sci-*φ* is an evolution of JJPF, our former proposed framework based on plain Java and using Jini to achieve automatic resource discovery and distributed task assignment. Computational resources are discovered and recruited automatically to compute user applications. Fault tolerance features have been included in the framework such that the execution of a parallel program can transparently resist node or network faults. Load balancing is guaranteed across the recruited computational resources, even in case of resources with fairly different computing capabilities. Sci-*φ* adds to JJPF several features. Some focusing on performance optimisations (especially when modern hardware is exploited), others aimed at providing its functionalities in the form of a Cloud PaaS solution. These are particularly interesting because they let Sci-*φ* be used as a building block for more complex parallel environments, like it happened to JJPF for PAL [23–25]. In the future we plan to adopt it again, possibly in different kinds of scenarios and environment. In order to evaluate the Sci-*φ* approach we conducted some preliminary experiments using a prototype we developed. Even if such prototype does not provide all the envisioned features characterising our Sci-*φ* solution, the preliminary results are nice and encouraging.

## Figures and Tables

**Algorithm 1:**
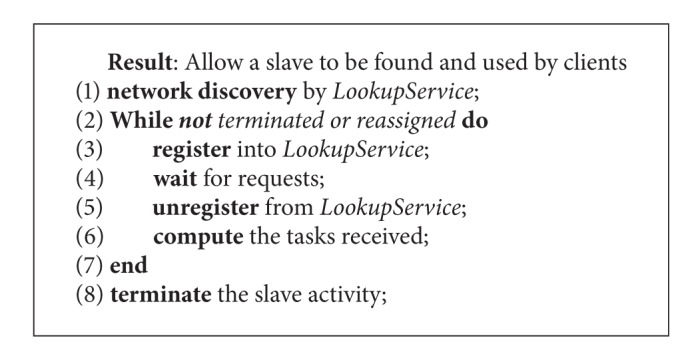
JJPF server side.

**Algorithm 2:**
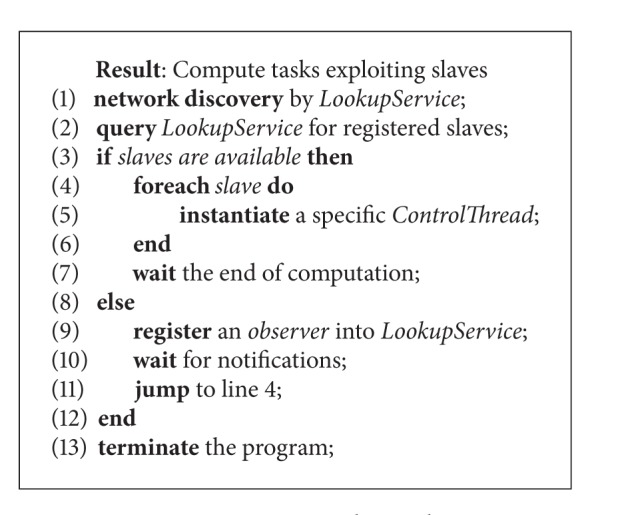
JJPF client side.
